# Effect of Alcohol Consumption Habits on Early Arterial Aging in Subjects with Metabolic Syndrome and Elevated Serum Uric Acid

**DOI:** 10.3390/nu15153346

**Published:** 2023-07-27

**Authors:** Alma Čypienė, Silvija Gimžauskaitė, Egidija Rinkūnienė, Eugenijus Jasiūnas, Aleksandras Laucevičius, Ligita Ryliškytė, Jolita Badarienė

**Affiliations:** 1 State Research Institute Centre for Innovative Medicine, 08406 Vilnius, Lithuania; alma.cypiene@santa.lt (A.Č.); aleksandras.laucevicius@santa.lt (A.L.); 2 Faculty of Medicine, Vilnius University, 03101 Vilnius, Lithuania; egidija.rinkuniene@santa.lt (E.R.); ligita.ryliskyte@santa.lt (L.R.); jolita.badariene@santa.lt (J.B.); 3 Center of Informatics and Development, Vilnius University Hospital Santaros Klinikos, 08661 Vilnius, Lithuania; eugenijus.jasiunas@santa.lt

**Keywords:** hyperuricemia, alcohol consumption, carotid–femoral pulse wave velocity, cardiovascular disease, metabolic syndrome, arterial stiffness

## Abstract

Background: Hyperuricemia is perceived as one of the risk factors for developing and progressing cardiovascular disease and metabolic syndrome through various pathological mechanisms. Endogenous synthesis and exogenous factors such as diet and beverages consumed play a major role in determining serum uric acid (sUA) levels. The aim of this study was to evaluate the effect of alcohol consumption on early arterial aging in middle-aged patients with metabolic syndrome (MetS) and hyperuricemia. Materials and Methods: This study included 661 middle-aged subjects (241 men and 420 women) from the Lithuanian High Cardiovascular Risk (LitHiR) primary prevention program. Characteristics of subjects such as blood pressure, laboratory testing, and the specialized nutrition profile questionnaire were evaluated. As an early marker of arterial stiffness, carotid–femoral pulse wave velocity (cfPWV) was assessed using a non-invasive applanation tonometry technique. Results: Hyperuricemia was present in 29% of men and 34% of women. Hyperuricemic men reported 1.6 times higher rates of alcohol drinking compared to men with normal sUA levels. After analyzing the correlation between alcohol consumption and cfPWV, no statistically significant relationships were found at a significance level of α = 0.05 but lowering the significance level to 0.06 revealed significant associations in men with normal sUA (ε^2^ordinal = 0.05, *p* = 0.06) and in women with increased sUA levels (ε^2^ordinal = 0.05, *p* = 0.08). Regression analysis showed that hyperuricemic men, consuming more than one unit of alcohol per week, had a significant impact on increasing cfPWV, while men with normal sUA levels, abstaining from alcohol entirely, resulted in a statistically significant decrease in cfPWV. Our results showed statistically significant relationships only among a group of men, although the women in the hyperuricemic group had a statistically higher cfPWV than women with normal sUA levels. Conclusions: Drinking alcohol is associated with increased arterial stiffness among hyperuricemic middle-aged men with MetS.

## 1. Introduction

Metabolic syndrome (MetS) is defined as a group of clinical and biological markers that include arterial hypertension (AH), central obesity, insulin resistance, hypertriglyceridemia, and low levels of high-density lipoprotein (HDL) cholesterol [1]. A new definition of MetS was presented in 2022, which recommended that MetS should comprise the presence of obesity and at least two of the following three criteria: high BP, impaired glucose metabolism, and elevated non-high-density lipoprotein (non-HDL) cholesterol level. According to the new approach, the authors have highlighted that MetS is not limited to the primary components only, but also encompasses additional conditions, such as hepatic steatosis, obstructive sleep apnea, heart failure with preserved ejection fraction, impaired kidney function, polycystic ovary syndrome, chronic inflammation, and hyperuricemia [2]. Despite the definition update, it is well known that all of these factors are interrelated and together increase the risk of developing cardiovascular disease (CVD) and type 2 diabetes [1].

Many studies have demonstrated that hyperuricemia, commonly defined in the literature as serum uric acid (sUA) levels greater than 430 μmol/L in men and greater than 360 μmol/L in women, increases the risk of developing MetS [3,4,5]. Nowadays, hyperuricemia can even be perceived as a manifestation or negative outcome of MetS [6]. Despite a growing body of research indicating a strong connection between elevated sUA concentration and MetS, the diagnostic criteria for MetS do not include this factor [7].

Nowadays, hyperuricemia is considered one of the risk factors for the development and progression of CVD, in addition to other well-known risk factors such as AH, diabetes, dyslipidemia, and obesity. Increased levels of sUA influence the onset of CVD, specifically coronary artery disease (CAD), through many different pathological mechanisms [8]. Atherosclerosis is known as an inflammatory disease and sUA itself induces inflammation by causing the production and activation of different inflammatory factors, also the extracellular signal-regulated kinase (ERK) and p38 mitogen-activated protein kinase (MAPK) cascade reaction [8,9,10]. Increased sUA levels also reduce nitric oxide (NO) production, which is one of the factors in the development of endothelial dysfunction, which is considered an early manifestation of the atherosclerosis process [11]. sUA also contributes to the dysregulation of the renin–angiotensin–aldosterone (RAAS) system by changing the expression of angiotensinogen, angiotensin-converting enzyme (ACE), and angiotensin II receptor levels. The imbalance of the RAAS system leads to the development of hypertension and atherosclerosis [12]. Xanthine oxidase (XO) activity rises during the synthesis of sUA, leading to an elevation in reactive oxygen species (ROS), which is one of the main reasons for oxidative stress [8,13]. On the contrary, sUA in adequate levels is also considered an antioxidant that helps to improve the response of endothelial cells to oxidative stress, thus reducing endothelial damage and mitigating atherosclerosis [14]. Nevertheless, abnormal sUA levels act through various pathways and early anti-hyperuricemic treatment can prevent the development of CAD [8].

Endogenous synthesis and exogenous factors such as diet and beverages consumed play a major role in determining the level of sUA. Consuming foods high in purines and excessive alcohol intake contribute to hyperuricemia by increasing sUA production and reducing its excretion [15]. Multiple cohort studies have found that individuals who consume alcohol have a 1.5- to 2-fold increased risk of developing hyperuricemia compared to those who do not consume alcohol. Although, the risk of developing gout and hyperuricemia may depend on the type of alcoholic beverage consumed [16]. Prospective study results showed that spirits lead to a lower risk of developing gout when compared to beer, while moderate consumption of wine does not increase the risk [17]. The reason for this difference can be attributed to the higher purine content of beer compared to other alcoholic beverages, with guanosine being the most relevant [18]. Furthermore, the metabolism of alcohol can lead to the production of lactate, which, as an anti-uricosuric agent, may inhibit the excretion of sUA by the proximal tubule and potentially exacerbate hyperuricemia [19].

Geographic location, as well as religious, cultural, and economic factors, determine both the quantity and variety of alcoholic beverages that are typically consumed in different parts of the world. Epidemiological studies showed that the consumption of beer was most prevalent in North America, South America, and Europe, while the consumption of spirits was highest in the Southeast Asia region, the Western Pacific, and the Middle East/Eastern Mediterranean region [20]. Although Lithuania has been placed among the top heavy-drinking countries for many years, recent data from The Lithuanian Drug, Tobacco, and Alcohol Control Department (NTAKD) showed that the impact of the Lithuanian alcohol policy model has resulted in a reduction in average annual alcohol consumption from 14.1 L per capita in 2018 to 12.1 L per capita in 2021 [21,22]. The high prevalence of alcohol consumption and its negative health effects highlight the need to identify populations with higher health priorities and implement targeted health interventions.

There is relatively little research specifically exploring the relationship between alcohol consumption, hyperuricemia, and the risk of arteriosclerosis within the context of MetS. For this study, we used carotid–femoral pulse wave velocity (cfPWV) as an early arteriosclerosis indicator to evaluate arterial stiffness. The European Society of Cardiology indicated that cfPWV values greater than 10 m/s are considered pathological [23]. Data from one meta-analysis showed that a 1 m/s increase in cfPWV was associated with a 1.12-fold increase in future CVD events [24]. By narrowing our investigation to the specific population, we aim to contribute valuable insights into potential interactions between alcohol intake, hyperuricemia, and atherosclerosis in individuals with MetS.

## 2. Materials and Methods

### 2.1. Study Population

Our study was carried out in the sub-department of preventive cardiology at VUH Santaros Klinikos between January 2018 and November 2019. The approval of the study protocol was given by Vilnius Regional Biomedical Research Ethics under reference number 158200-18/4-1006-521. The study utilized data from the Lithuanian High Cardiovascular Risk (LitHiR) primary prevention program, which began in 2006 with the aim of preventing early atherosclerosis development in high cardiovascular risk patients. The program focuses on identifying cardiovascular risk factors in primary prevention patients with MetS, and, through appropriate modifications, aims to reduce mortality from CVD.

This study’s participants included female patients aged 50–65 years and male patients aged 40–55 years who had been diagnosed with MetS. MetS was determined using the modified National Cholesterol Education Program Adult Treatment Panel III (NCEP ATP III) criteria, when at least 3 of the 5 risk factors were present: (1) serum triglyceride (TG) levels ≥ 1.7 mmol/L; (2) HDL cholesterol < 1.03 mmol/L for men and <1.29 mmol/L for women; (3) waist circumference ≥ 102 cm for men and ≥88 cm for women; (4) BP equal to or greater than 130/85 mmHg or currently undergoing anti-hypertensive treatment; and (5) fasting plasma glucose ≥ 5.6 mmol/L. Patients were excluded from the analysis if they had a previously diagnosed CAD, stroke, peripheral artery disease, oncological disease, advanced kidney or hepatic failure, chronic arrhythmias, severe psychiatric disorders, pregnancy, drug addiction, gout, and treatment with xanthine oxidase inhibitors. The specific age range of patients differed due to the similarity in CVD risk between men aged 50 years and women aged 60 years [25,26].

### 2.2. Evaluation of the Study Population

The study protocol adhered to obtaining participants’ written consent forms before conducting any research procedures. Clinical examinations were conducted in a peaceful and comfortable room with temperatures maintained at 22–24 °C during mornings after a 12 h period of fasting and abstaining from alcohol and beverages containing caffeine. Physical measurements were taken, including weight, height, waist circumference, body mass index (BMI), and BP. BP was measured manually using the Riester precisa^®^ N Sphygmomanometer, following the 2018 European Society of Cardiology Guidelines for the Management of Arterial Hypertension [23]. Blood samples were collected to assess concentrations of sUA, serum creatinine, TG, serum total cholesterol, LDL cholesterol, HDL cholesterol, and high-sensitivity C-reactive protein (hs-CRP) and were analyzed using the “Abbott Architect ci8200 PLUS” (Abbott Laboratories, Chicago, IL, USA) analyzer. Hyperuricemia was determined when the sUA level exceeded 357 µmol/L in females and 428 µmol/L in males, and patients were categorized based on its presence or absence.

Arterial stiffness was assessed by evaluating carotid and femoral pulse waves in the supine position using a non-invasive means of the applanation tonometry technique (Sphygmocor v.7.01, AtCor Medical Pty. Ltd. 1999–2002, Sydney, Australia). The pulse wave transit time was measured and determined as cfPWV. The mean blood pressure (MBP) was calculated automatically using the formula that MBP equals the sum of 1/3 systolic blood pressure (SBP) and 2/3 diastolic blood pressure.

A specialized questionnaire was used to gather information on participants’ place of residence, education, marital status, drug usage, smoking habits, and dietary patterns, including meat, dairy, and fish consumption, water intake, and alcohol consumption. In this particular study, we emphasized alcohol intake. The participants were asked to report their alcohol intake in units per week for the previous week, with options ranging from less than one, one, more than one unit, to no alcohol consumption at all. In the survey, a single unit of alcohol was explained to be equivalent to 80 mL of wine, 250 mL of beer, or 30–50 mL of spirits. Efforts were made to clarify the given information by repeatedly contacting the participants.

### 2.3. Statistical Analysis

For the statistical analysis, the R statistical software package V.4.0.2, RStudio V.1.3.959, IBM SPSS Statistics V.23, and G*Power V.3.1.9.4 were used.

Descriptive statistics were used to describe interval and ratio variables in terms of medians, first quartiles (Q1), and third quartiles (Q3), while the normality of data was checked using Shapiro–Wilk and Kolmogorov–Smirnov (K–S) tests. Categorical variables were described using percentages and frequencies. Linear regression equations were used to build models to determine the influence of independent variables on the dependent variable. The Breusch–Pagan test was used to check for heteroscedasticity, and Pearson’s chi-squared test to determine the statistically significant difference among the independent groups. Spearman’s correlation coefficient (rS) was used to determine the effect size between interval variables, where an effect size was considered low if 0.1 ≤ rS < 0.3, moderate if 0.3 ≤ rS < 0.5, and large if rS ≥ 0.5.

To measure the effect between normally distributed interval variables, we used the omega squared partial (ω2p) effect size, and to evaluate the effect size between interval variables that do not meet the condition for normal distribution, we used the rank epsilon squared ordinal (ε^2^ordinal) effect size. Effect sizes were classified as small if 0.01 ≤ ε^2^ordinal (ω2p) < 0.06, moderate if 0.06 ≤ ε^2^ordinal (ω2p) < 0.14, and large if ε^2^ordinal (ω2p) ≥ 0.14.

To determine statistical significance, we used a *p*-value < 0.05.

## 3. Results

In this cross-sectional study, 661 patients were evaluated. The majority of study participants were women (66%), aged between 50 and 65 years, while the remaining portion consisted of men between the ages of 40 and 55. Male and female participants were separated into two categories of normal and elevated sUA levels (29% of men and 34% of women with hyperuricemia).

For men, statistically significant dependencies were found between BMI, waist circumference, systolic blood pressure (SBP), fasting glucose, and creatinine levels. In contrast, statistically significant dependencies for women were found between BMI, SBP, HDL cholesterol, TG, hs-CRP, and creatinine levels. However, when comparing the two groups of male and female patients based on their education, marital status, place of residence, use of drugs, and smoking status, no significant differences were found between those with increased sUA and normal sUA levels (Table 1).

In our analysis, we observed a statistically significant difference in alcohol consumption among the male participants with normal and elevated sUA levels. Men with normal sUA levels reported their alcohol intake more frequently as less than one alcohol unit per week or no drinking of alcoholic beverages at all, while the analysis showed 1.6 times higher rates of drinking more than one unit of alcohol per week among males with hyperuricemia compared to men with normal sUA levels.

However, no notable disparities in alcohol intake patterns were detected among the female subjects (Table 2).

In this study, we compared cfPWV, a measure of aortic stiffness, between male and female participants with normal and elevated sUA levels. Hyperuricemic women showed a greater cfPWV compared to those with normal sUA levels (*p* = 0.004). On the other hand, there was no significant difference in cfPWV among men, although men with elevated sUA levels had a substantially higher applanation tonometry-derived mean blood pressure (MBP) (Table 3).

**Figure 1:**
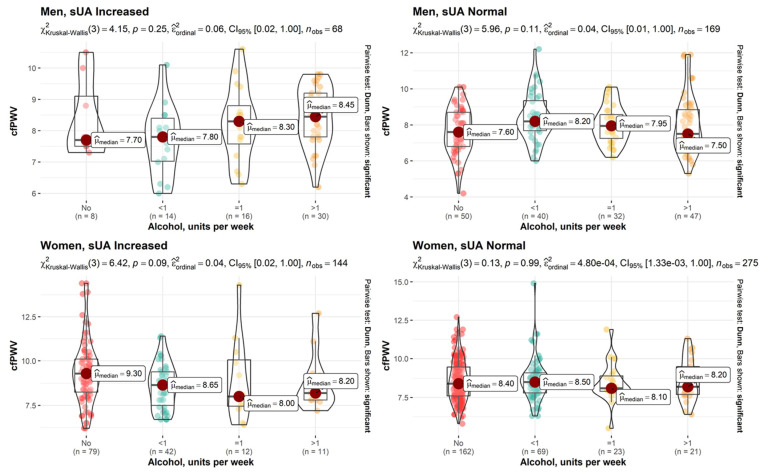
Effect sizes between weekly alcohol consumption and cfPWV for male (upper row) and female (lower row) study subjects with elevated and normal sUA levels.

Further in our study, a correlation analysis was conducted. After analyzing the impact of weekly alcohol consumption on cfPWV at a significance level of α = 0.05, no statistically significant relationships were found. Nonetheless, when the significance level was lowered to 0.06, we observed quite significant associations in men with normal sUA (ε^2^ordinal = 0.05, *p* = 0.06) and in women with increased sUA levels (ε^2^ordinal = 0.05, *p* = 0.08) (Figure 1).

Finally, in our study, we developed regression equations containing all the indicators presented in Table 1 and Table 2 to assess the influence of specific risk factors on cfPWV between study subjects with normal and elevated sUA levels. We determined that the linear regression equation provided the most accurate prediction of cfPWV. The cfPWV histogram was also observed to have a bell shape. All regression equations demonstrated a strong fit with the data, with coefficients of determination (R^2^) greater than 0.20 for each equation.

**Figure 2:**
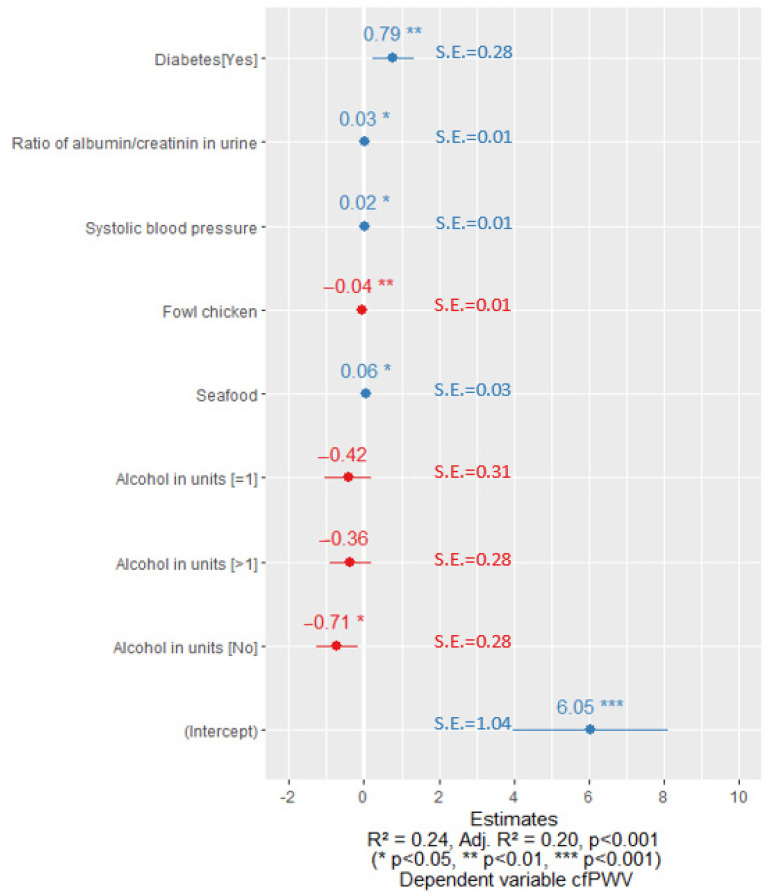
Linear regression coefficients for men with normal sUA levels. Blue color represents the positive value of the coefficients and the red color represents the negative value of the coefficients. S.E.—standard error.

**Figure 3:**
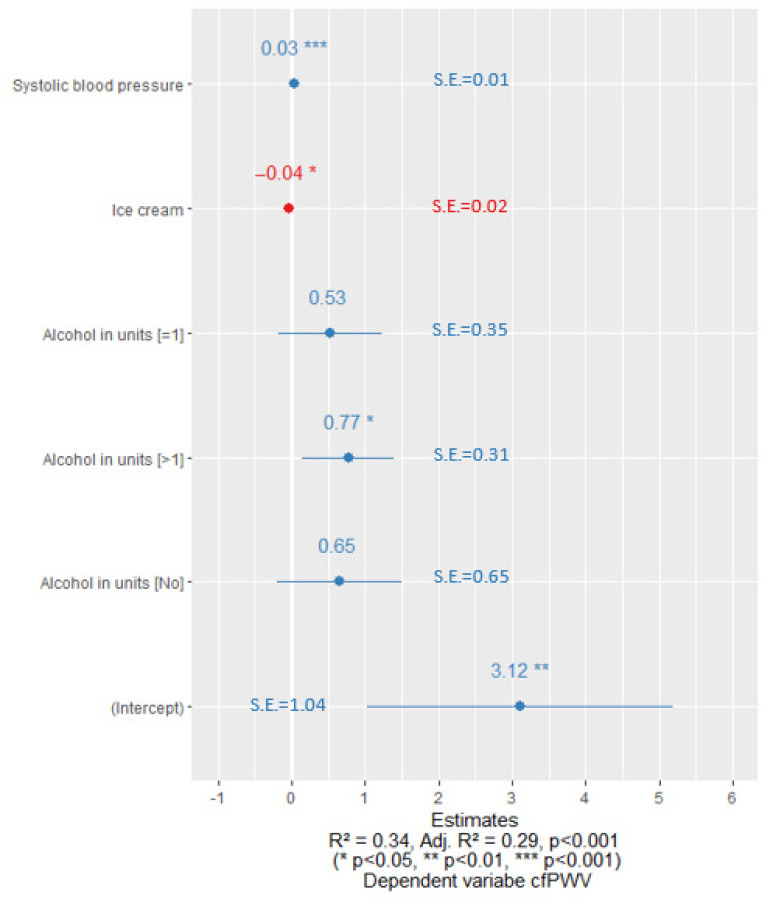
Linear regression coefficients for men with increased sUA levels. Blue color represents the positive value of the coefficients and the red color represents the negative value of the coefficients. S.E.—standard error.

After optimizing the regression equations, we observed that the cfPWV was significantly affected by alcohol consumption habits. In the case of men with normal sUA levels, if they did not consume alcohol along with other risk factors, there was a statistically significant decline in cfPWV as shown in Figure 2. In contrast, in a sub-group of men with elevated sUA levels, drinking more than one unit of alcohol per week in combination with other risk factors showed a statistically significant association with increased cfPWV (Figure 3).

## 4. Discussion

This retrospective study aimed to assess the association between hyperuricemia and alcohol consumption, and their influence on early vascular aging.

The results of our study indicated that hyperuricemia was present in 29% of male patients and 34% of female patients in a primary prevention setting. These patients also showed a higher incidence of different CVD risk factors, such as a significantly higher BMI, waist circumference, and SBP. The BP-CARE study analyzed hypertensive patients without overt CVD from a geographical background similar to that of Lithuania and exhibited comparable results. Specifically, 23.5% of males and 28% of females were classified as hyperuricemic and showed a higher prevalence of CVD risk factors [27]. Nevertheless, there are differences in the prevalence of hyperuricemia among various countries, with around 15% in Italy or the US, 16.4% in the Chinese population, 25% in the Philippines, 10–52% in Taiwan, and as high as 85% in the Marshall Islands [28,29,30,31]. The variations in the prevalence of hyperuricemia among different populations may be attributed to many genetic and lifestyle factors, such as obesity and dietary habits, including the consumption of foods high in purine or alcoholic beverages [27]. An analysis of National Health and Nutrition Examination Survey (NHANES) data revealed that elevated sUA levels were associated with BMI, DASH diet (Dietary Approaches to Stop Hypertension diet, which helps to lower salt in diets and, thus, reduce BP), diuretic use, and alcohol consumption, which may be considered the main modifiable factors in the development of hyperuricemia [32].

The metabolism of sUA is influenced by the balance between urate synthesis and excretion. Alcohol plays a role in this process by increasing lactate levels. Alcohol dehydrogenase causes the reduction of the coenzyme NAD+ to NADH, which subsequently stimulates the conversion of pyruvate to lactate through lactate dehydrogenase. The resulting lactate anion then enhances the activity of the URAT1 transporter, leading to increased reabsorption of filtered urate [33]. Thus, there is a direct link between alcohol consumption and urate metabolism, and modifying alcohol intake can result in a reduction in sUA levels.

The link between alcohol consumption and hyperuricemia, particularly its most common clinical manifestation, gout, has been investigated in many studies [17,34,35,36]. Our analysis showed that men with elevated sUA levels reported higher rates of alcohol drinking. Results from a prospective study that lasted more than 12 years and involved a large cohort of men showed that even low amounts of alcohol (less than 14 g/day) increase the risk of gout. Furthermore, this risk increases even further with higher rates of alcohol consumption. Notably, this particular association was independent of other risk factors such as age, dietary habits, BMI, AH, use of diuretics, or chronic kidney disease [35].

In our study, we analyzed the association between alcohol consumption and arterial stiffness, which is considered an indicator of early sub-clinical atherosclerosis, in individuals with both normal and elevated sUA levels. Our regression analysis showed that among men with hyperuricemia, consuming more than one unit of alcohol per week had a significant impact on the worsening arterial stiffness parameter, cfPWV. Conversely, in men with normal sUA levels, abstaining from alcohol entirely resulted in a statistically significant decrease in cfPWV. Although the women in the hyperuricemic group had a statistically higher cfPWV, our study showed no statistically significant relationships between cfPWV and alcohol intake. This observation may be attributed to the higher frequency and larger quantities of alcohol consumption among men compared to women. Also, women tend to underreport or withhold information about their alcohol consumption, which might have influenced the responses to the questionnaire.

In a systematic review conducted by R Del Giorno et al., heterogeneous results were observed concerning the quantity of alcohol consumed and its influence on arterial stiffness. While many studies agreed on similar results that heavy alcohol consumption (e.g., exceeding 70 g per week) is associated with higher values of cfPWV, several studies indicated a J-shaped relationship between alcohol consumption and cfPWV. This revealed that moderate alcohol intake is associated with lower cfPWV values compared to abstaining from alcohol completely or consuming excessive amounts [37].

In 2020, alcohol consumption rates in Lithuania were high, with an average intake ranging from five to seven standard units of alcohol, when one standard unit of alcohol per day was defined as 10 g of pure alcohol [38]. In the 2021 ESC Guidelines on cardiovascular disease prevention in clinical practice, it was recommended not to exceed 100 g of pure alcohol per week for both men and women. While the data were suggesting that moderate alcohol consumption is associated with a lower risk of CVD, Mendelian randomization studies have shown that abstainers have the lowest risk of developing CVD. Furthermore, even the smallest amounts of alcohol have been found to increase BMI and BP [39]. Although the rates of hazardous alcohol consumption in Lithuania, defined as consuming 60 g or more of pure alcohol on a single occasion, have decreased from 56% to 38% since 2014, alcohol consumption remains a significant health concern [38].

However, we must acknowledge several potential limitations of our study. Firstly, it was based on a cross-sectional design; thus, no causal effects may be confirmed. Randomized controlled studies with larger study samples are still required. Secondly, alcohol consumption was evaluated using a self-reported survey, which may introduce subjectivity when reporting alcohol usage rates, especially if the consumption is high. Finally, we did not consider the impact of various types of alcoholic beverages on hyperuricemia and arterial stiffness, which could have revealed different effects on vascular health and thus, could have been relevant for population-based strategies.

## 5. Conclusions

Our study further strengthened the evidence linking alcohol consumption to increased arterial stiffness. The findings demonstrated that among hyperuricemic men, consuming more than one unit of alcohol per week significantly increased cfPWV. Further research is required to analyze the effect of alcohol on hyperuricemia and vascular health in patients with MetS, aiming to improve population-based recommendations for the management of hyperuricemia and CVD risk in a broader context.

## Figures and Tables

**Table 1:** Clinical characteristics of the study subjects.

Variable	Men (N = 241)			Women (N = 420)			Overall
	sUA Elevated (N = 70)	sUA Normal (N = 171)	*p*-Value	sUA Elevated (N = 144)	sUA Normal (N = 276)	*p*-Value	
Age, years			<0.05			0.51	
Mdn [Q1, Q3]	46.0 [43.0, 48.0]	48.0 [44.0, 51.0]		58.0 [54.0, 61.0]	58.0 [54.0, 61.0]		54.0 [49.0, 59.0]
BMI, kg/m^2^			<0.05			<0.05	
Mdn [Q1, Q3]	31.7 [30.1, 35.1]	30.5 [28.1, 32.8]		33.6 [30.1, 37.5]	30.5 [27.4, 33.6]		31.2 [28.4, 34.3]
Circumference of waist, cm			<0.001			0.06	
Mdn [Q1, Q3]	109 [104, 113]	105 [102, 110]		105 [97.0, 112]	97.0 [91.0, 105]		103 [95.0, 109]
SBP, mmHg			0.05			0.04	
Mdn [Q1, Q3]	139 [128, 148]	133 [126, 142]		137 [130, 146]	135 [123, 145]		135 [126, 145]
sUA, µmol/L			<0.05			<0.05	
Mdn [Q1, Q3]	481 [457, 520]	366 [328, 393]		397 [378, 447]	291 [259, 324]		352 [298, 404]
LDL-cholesterol, mmol/L			0.37			0.36	
Mdn [Q1, Q3]	3.81 [3.14, 4.28]	3.60 [2.92, 4.25]		3.67 [3.06, 4.36]	3.89 [3.01, 4.83]		3.74 [2.98, 4.49]
TG, mmol/L			0.44			<0.001	
Mdn [Q1, Q3]	2.39 [1.59, 2.83]	1.82 [1.26, 2.53]		1.79 [1.37, 2.43]	1.52 [1.07, 2.03]		1.73 [1.24, 2.39]
Fasting glucose, mmol/L			0.04			0.39	
Mdn [Q1, Q3]	5.98 [5.61, 6.51]	6.03 [5.63, 6.49]		6.13 [5.79, 6.94]	6.00 [5.66, 6.54]		6.06 [5.66, 6.60]
hs-CRP, mg/L			0.09			<0.001	
Mdn [Q1, Q3]	1.99 [1.16, 3.91]	1.46 [0.783, 2.35]		2.61 [1.15, 4.62]	1.46 [0.810, 2.76]		1.60 [0.880, 3.10]
Creatinine, μmol/L			<0.001			<0.001	
Mdn [Q1, Q3]	81.5 [77.0, 91.0]	77.0 [71.5, 84.0]		68.0 [62.0, 73.0]	65.0 [60.0, 70.0]		70.0 [63.0, 78.0]
Diabetes			0.92			0.07	
Yes	11.0 (15.7%)	26.0 (15.2%)		38.0 (26.4%)	52.0 (18.8%)		127 (19.2%)
No	59.0 (84.3%)	145 (84.8%)		106 (73.6%)	224 (81.2%)		534 (80.8%)

BMI—body mass index, hs-CRP—high-sensitivity C-reactive protein, LDL-cholesterol—low-density lipoprotein cholesterol, Mdn—median, SBP—systolic blood pressure, sUA—serum uric acid, TG—triglycerides.

**Table 2:** Alcohol consumption habits of study participants.

Variable	Men (N = 241)			Women (N = 420)			Overall
	sUA Elevated (N = 70)	sUA Normal (N = 171)	*p*-Value	sUA Elevated (N = 144)	sUA Normal (N = 276)	*p*-Value	
Alcohol, units per week			0.012			0.83	
<1	14 (20.6%)	40 (23.7%)		42 (29.2%)	69 (25.1%)		165 (25.2%)
1	16 (23.5%)	32 (18.9%)		12 (8.33%)	23 (8.36%)		83 (12.7%)
>1	30 (44.1%)	47 (27.8%)		11 (7.64%)	21 (7.64%)		109 (16.6%)
No	8 (11.8%)	50 (29.6%)		79 (54.9%)	162 (58.9%)		299 (45.6%)
Missing	2 (2.9%)	2 (1.2%)		0 (0%)	1 (0.4%)		5 (0.8%)

sUA—serum uric acid.

**Table 3:** The characteristics of cfPWV and applanation tonometry-derived MBP in the study participants.

Variable	Men (N = 241)			Women (N = 420)			Overall
	sUA Elevated (N = 70)	sUA Normal (N = 171)	*p*-Value	sUA Elevated (N = 144)	sUA Normal (N = 276)	*p*-Value	
cfPWV, m/s			0.14			0.004	
Mdn (Q1, Q3)	8.15 (7.53, 8.98)	8.00 (7.00, 8.75)		8.90 (7.80, 9.90)	8.40 (7.70, 9.33)		8.40 (7.60, 9.30)
MBP, mmHg			0.05			0.519	
Mdn (Q1, Q3)	101 (95.0, 109)	99.0 (93.0, 106)		99.0 (94.0, 106)	99.0 (91.8, 106)		99.0 (93.0, 106)

cfPWV—carotid–femoral pulse wave velocity, MBP—mean blood pressure, Mdn—median, sUA—serum uric acid.

## Data Availability

The study data are available on request from the corresponding author.
